# Fast Detection of Defective Insulator Based on Improved YOLOv5s

**DOI:** 10.1155/2022/8955292

**Published:** 2022-09-03

**Authors:** Zhao Liquan, Zou Mengjun, Cui Ying, Jia Yanfei

**Affiliations:** ^1^Key Laboratory of Modern Power System Simulation and Control & Renewable Energy Technology, Ministry of Education (Northeast Electric Power University), Jilin 132013, China; ^2^Guangdong Electric Power Corporation, Zhuhai Power Supply Bureau, Zhuhai 519000, China; ^3^College of Electrical and Information Engineering, Beihua University, Jilin 132013, China

## Abstract

Defective insulator detection is an essential part of transmission line inspections based on unmanned aerial vehicles. It can timely discover insulator defects and repair them to avoid a power transmission accident. The detection speed of defective insulators based on artificial intelligence directly affects inspection efficiency. To improve the detection speed of defective insulators based on YOLOv5s, an improved detection method with faster detection speed and acceptable precision is proposed. First, a new ResNet unit with three branches is designed based on depthwise separable convolution with kernel three and average pooling. To reduce parameters, the new ResNet unit is used to replace the original ResNet unit used in the CSP1_X module in YOLOv5s. Besides, we also introduce channel shuffle in the CSP1_X module to facilitate the flow of feature information from different channels. Second, a new residual CBL module is designed based on depthwise separable and standard convolution. The new residual CBL module is used to replace the two CBL modules used in the CSP2_X module in YOLOv5s to reduce parameters and extract more useful features. Third, we design a separate, coordinated attention module by introducing location information into channel attention. The new attention module is added to the end of the CSP2_X module to improve the ability to extract insulator location information. Besides, we also use convolution to replace the focus model to reduce computation. Compared with defective insulator detection methods, the proposed method has smaller parameters, floating-point operations per second, and higher frames per second. Although it has lower mean precision, it has a faster detection speed. Besides, the increase in detection speed is greater than the decrease in mean precision.

## 1. Introduction

Unmanned aerial vehicle has been widely used in transmission line inspection to improve inspection efficiency and reduce the workload for inspectors [1, 2]. In the traditional transmission line inspection based on an unmanned aerial vehicle, the inspector detects the defective transmission devices by watching the screen. It takes a lot of time to detect faulty devices, which is affected by the screen size and the light intensity. With the development of artificial intelligence technology, the deep learning method has been widely used in object tracking [3, 4], image super-resolution reconstruction [5], image dehazing [6], and defective transmission device detection [7]. The images of transmission line devices captured by the unmanned aerial vehicle are transmitted via a wireless mobile communication network to a server with high computing power. The captured images are detected by artificial intelligence methods deployed on the server. It consumes much time to transmit images to serves, affecting transmission devices' detection speed. It cannot be used in detection areas without wireless mobile network coverage.

With the development of portable edge computing devices, the captured images can be detected by artificial intelligence methods deployed on the mobile edge computing device [8]. The captured images are transmitted to portable edge computing devices carried by inspectors with short-range wireless communication. It is not dependent on the mobile network and has a greater range of applications. The computation power of portable edge computing devices is limited, which affects the detection speed. In order to improve detection speed, one is to improve the computation power of portable edge computing devices, and the other is to reduce the computation of the artificial intelligence methods. The cost of reducing computation is lower. Therefore, we mainly focus on how to reduce computation.

Object detection based on deep learning can be divided into two categories: one-stage detection method and two-stage detection method. In the one-stage method, it directly generates detection boxes and classifies the objects without generating region proposals. The two-stage method divides the detection process into two stages. It generates region proposals in the first stage, regresses the bounding box and candidate regions, and classifies the objects in the second stage. The one-stage method focus on improving detection speed. The two-stage method focus on improving detection precision. The two-stage method is mainly developed on the serves with high computing power. It is more suitable for offline object detection. The one-stage method has lower computation than two-stage method. It is more suitable for online object detection. Although it has been widely used in defective insulator detection, the detection is still required to be improved to meet the practical requirements.

YOLOv5 is one of the one-stage methods [9]. Although it is named YOLOv5, it is designed based on YOLOv3 [10] and is unrelated to YOLOv4 [11]. The YOLOv5 has four versions that are YOLOv5s, YOLOv5m, YOLOv5l, and YOLOv5x. The YOLOv5s has a smaller computation and lower precision. The YOLOv5x has a larger computation and higher precision. The YOLOv5s is more suitable for deploying on the portable edge computing device. To improve the defective insulator detection speed of YOLOv5s with acceptable precision, we propose an improved YOLOv5s.

For this paper, the main contributions are as follows:

1. To reduce computation and improve the detection speed of defective insulators, we design a new ResNet unit with two branches, and use it to replace the original ResNet unit used in CSP1_X module in YOLOv5s. The new ResNet is based on depthwise separable convolution with kernel three and average pooling. It has smaller computation than the original ResNet unit In YOLOv5s. Besides, to extract more useful features from different channels, we also introduce the channel shuffle operation into CSP1_X module in YOLOv5s.
2. To further reduce the computation, we also design a residual CBL module and use it instead of two original CBL modules in CSP2_X module in YOLOv5s. The residual CBL module has smaller computation than the original two CBL modules. Besides, to extract more useful features from different channels, we also introduce the channel shuffle operation into CSP2_X module in YOLOv5s to facilitate the flow of feature information from different channels.
3. To reduce background interference and make the network pay more attention to useful information, we design a separate embedded coordinated attention module and introduce it into the CSP2_X module to extract more useful features. Besides, to reduce computation, we also use convolution with kernel 3 to replace the focus.

The traditional transmission line inspection based on unmanned aerial vehicle requires a human eye view of the defective insulators. To improve the inspection efficiency based on unmanned aerial vehicle, artificial intelligence methods are deployed on servers or ground service stations to automatically detect defective insulators from the captured image by unmanned aerial vehicles. Although many artificial intelligence methods have higher precision, they have slower detection speed that directly affects inspection efficiency. To improve defective insulator detection speed with acceptable detection precision, we propose an improved YOLOv5s with lower computation for defective insulator detection to improve the inspection efficiency. The structure of the rest of this article is organized as follows. Related work is provided in Section 2. Our proposed method are introduced in Section 3. The experimental results and discussions are reported in Section 4. Finally, conclusions are given in Section 5.

## 2. Related Work

Object detection method based on deep learning has been widely used in defective insulator and transmission device detection. There are two categories of object detection models. The first category is two-stage detection models, such as Faster R–CNN [12], Mask R–CNN [13], Cascade R–CNN [14], and Sparse R–CNN [15]. The second category is one-stage detection models, such as the SSD series method [16, 17] and the YOLO series method [9–11]. The two-stage has a larger computation and is more suitable for offline detection. The one-stage has smaller computation and is more suitable for online detection. Therefore, the defective insulator detection method based on a one-stage method is more suitable for developing a portable edge computing device with limited computation power.

The YOLO series method is the most representative method of the one-stage detection method. The YOLOv1method was first proposed by Redmon et al. [18]. It creatively combined candidate area and object recognition into one stage. It transformed the object recognition problem into a regression problem and directly predicted the location and class of the object using a depth convolution neural network. Compared with the two-stage method, it has lower computation and acceptable accuracy. Due to these advantages, the YOLO method attracts much attention from many scholars and has become an important branch of object detection research based on deep convolution neural networks. To improve the detection speed and precision of YOLOv1, Redmon et al. [19] proposed YOLOv2. In YOLOv2, batch normalization is introduced into the convolution layer. The new convolution layer consists of convolution, batch normalization, and LeakyReLU activating function. In the VOC2007 dataset, the mean average precision is improved from 63.4% to 65.8%. In order to solve the problem caused by the different training and detection image sizes, YOLOv2 fine-tuned the classification network that had been trained on 244 × 244 low-resolution images on 448 × 448 high-resolution images. After fine-tuning, the final global average pooling and softmax layers are removed as the final backbone network. Then mean average precision is improved from 65.8% to 69.5%. It also introduced anchor boxes inspired by Faster R–CNN to improve precision. The size of the output feature map is 13 × 13. Each cell contains five anchor boxes to predict five bound boxes. Besides, YOLOv2 designed a new network named Darket-19 and used it as a backbone. In 2018, Redmon proposed YOLOv3, which is also the last version proposed by Redmon [10]. In order to extract more useful features, YOLOv3 used the Darknet-53 as the backbone and introduced feature pyramid networks to fuse more features. The number of network layers increased and contained many residual networks in Darknet-53. Besides, it also proposed binary cross-entropy loss for classification. Although Redmon withdraws from research in artificial intelligence, many improved YOLOv3 are proposed by scholars.

Yang et al. proposed GC-YOLOv3 based on YOLOv3 to improve the mean average precision [20]. They designed a cascading network that consisted of learnable semantic fusion and used a global self-attention mechanism to extract more useful information. Qu et al. proposed an improved YOLOv3 with auxiliary networks for remote sensing image detection to improve detection precision and detection speed [21]. They used an image blocking module to feed fixed images and DIOU to replace IOU in YOLOv3. Besides, they used a convolutional block attention module to connect the backbone network and designed an auxiliary network. In order to improve detection speed, the adaptive feature fusion method was also introduced into the improved YOLOv3. In order to improve detection speed, Yin et al. proposed Faster-YOLO [22]. They used ELELEM-AE joint network and DRKCELM network to design a feature extractor. The feature extractor integrates the advantages of ELM-EA and ELM-LRF. The detection speed of Faster-YOLO is two times faster than YOLOv3. In order to improve detection precision, Cai et al. proposed a modified YOLOv3 [23] based on MobileNetv1. They used the MobileNetv1 to replace the backbone of YOLOv3 and optimized the feature map size according to the detection results.

In 2020, Alexey et al. [11] proposed the YOLOv4 method base on YOLOv3. They used the cross-stage partial connections network to replace the residual block in YOLOv3 to design the backbone of YOLOv4. The path aggregation network also was used to fuse more features. They used spatial pyramid pooling to realize multi-scale features fusion and mish function to replace the LeakyReLu function as a new activate function. Besides, they used the mosaic data argument method to improve detection precision. The YOLOv4 has a faster detection speed and precision. To improve the detection speed of YOLOv4, Deng et al. [24] proposed an improved YOLOv4. They used feature pyramid networks and atrous spatial pyramid pooling to modify the MobileNetV3 to improve real-time and feature extraction ability. The improved MobileNetV3 is used as the backbone of YOLOv4 to reduce computation. They also introduced a convolutional block attention module to YOLOv4 to extract more useful features. The original team of YOLOv4 proposed scaled-YOLOv4 based on YOLOv4-CSP to make the model can be developed on different devices [25]. To further improve the performance of YOLOv4, some improved methods based on YOLOv4 have also been proposed [26–28]. To improve the one-stage method performance, Glenn proposed YOLOv5 based on YOLOv3 [10]. YOLOv5 designed two types of cross-stage partial (CSP) Networks that are CSP1_X and CSP2_X. The CSP1_X modules were used in the backbone, and the CSP2_X were used in the Neck part of YOLOv5. YOLOv5 has four different version networks that are YOLOv5s, YOLOv5m, YOLOv5l, and YOLOv5x. The different version of YOLOv5 has different depth CSP modules. YOLOv5 used the LeakyReLU function to activate the function in middle/hidden layers and the sigmoid function as an activated function in detection layers. Besides, YOLOv5 used GIOU loss as the loss function of the bounding box. YOLOv5s has a faster detection than YOLOv4 andYOLOv3. It is more suitable for developing portable edge computing devices.

Due to the advantage of the YOLO series method, they have been widely used in transmission devices and defective insulator detection. Liu et al. [29] proposed an aerial insulator image detection method based on YOLOv3 for high-voltage transmission lines. A cross-stage partially densely connected module was proposed to solve the feature reuse and propagation of feature layers in low-resolution images. It had higher detection precision defective insulator detection in complex transmission line backgrounds than YOLOv3 and YOLOv4. Qiuet al. [30] proposed a defective insulator detection algorithm based on YOLOv4. They used the Graph Cut data enhancement method to produce a new dataset, and the Laplace sharpening method was used to preprocess the insulator dataset images. To make the algorithm more lightweight, they used MobileNet as the backbone network of YOLOv4. It had a faster detection speed than YOLOv4. He et al. [31] proposed a self-exploding insulator detection algorithm based on YOLOv4. They proposed a new feature fusion structure and an improved SE attention mechanism, which effectively suppressed useless features and achieved higher detection accuracy than YOLOv4. Feng et al. [32] verified in detail the performance of YOLOv5s, YOLOv5m, YOLOv5l, and YOLOv5x on the public dataset China Power Line Insulation Dataset (CPLID), and the experimental results showed that YOLOv5 performed well on this dataset, especially YOLOv5x, with a detection accuracy of 95.5%, which can effectively identify defective insulators. Since the YOLOv5x model has a large number of parameters, Lan et al. [33]selected YOLOv5s with a smaller number of parameters as the baseline model, making it lighter by replacing the original CSP structure with the Ghost module and adding CBAM attention to improve the detection accuracy. To further improve the detection accuracy of defective insulators, some other methods based on YOLO have also been proposed [34–36].

## 3. Improved YOLOv5s

### 3.1. Improved CSP Module

Two types of CSP modules are given as CSP1_X and CSP2_X in YOLOv5s. The CSP1_X module is used in the backbone to extract features and the CSP2_X module is used in the Neck to fuse features. The CSP1_X consists of two branches. The first branch mainly consists of CBL module, ResNet units, and a convolution layer. The second branch only consists of a convolution layer. The output features of two branches are fused by concatenate operation. The ResNet unit computation directly affects detection speed.

**Figure 1:**
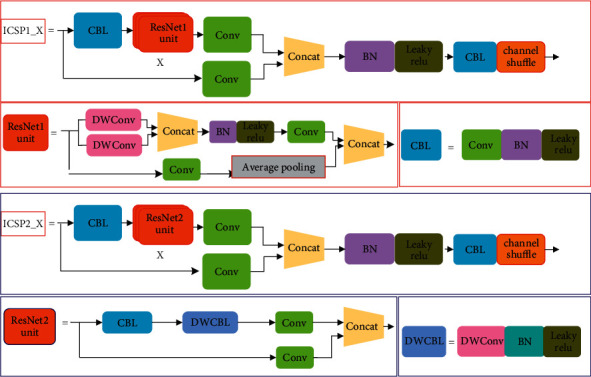
Improved CSP1_X and CSP2_X.

To reduce the computation of CPS1_X, we designed a new ResNet unit and replaced the original ResNet unit in CSP1_X. The proposed ResNet unit is named as ResNet1 unit and is shown in Figure 1. The proposed ResNet1 unit consists of two branches that we name the upper branch and lower branch, respectively. The upper branch contains two parallel depthwise separable convolutions (DWConv). The kernel sizes of the two depthwise separable convolutions are 3, and the number of output channels is half the number of input channels. We use the two parallel depthwise separable convolutions to extract different features. Therefore, we use concatenate operation to connect the output feature maps of two DWConv to keep the number of channels constant. To avoid gradient explosion or gradient disappearance and improve training speed, we add batch normalization (BN) and LeakyReLU activating function in the upper branch. Besides, we use a 1 × 1 convolution to reduce the number of output channels to halve the original number. The lower branches contain a 1 × 1 convolution and an average pooling. The 1 × 1 convolution is used to reduce the number of channels to make the lower branch have the same number of channels as the upper branch.

The average pooling is used to reduce the value of the background feature, which is useful for separating the defective insulator and background information. In the end, the upper and lower branches are connected by concatenating operation to construct the complete ResNet1 unit. We use the proposed ResNet1 unit to replace the original ResNet unit to reduce the computation of CSP1_X. In the end, we use the BN module, LeakyReLU activating function, and CBL module to extract features from the concatenated feature map. The CBL module consists of a 1 × 1 standard convolution, a BN module, and a LeakyReLU activating function. They are used to increase network depth to make the network extract more detailed information about the defective insulator. The improved CPS1_X is named ICPS1_X which is shown in Figure 1. The *X* is the number of ResNet1 unit used in ICSP1_X. The *X* is different in different modules. In YOLOv5s, two different ICSP1_X modules are present, i.e., ICSP1_1 and ICSP1_3. The numbers of ResNet1 unit of ICSP1_1 and ICSP1_3 is 1 and 3, respectively. The ICSP1_X module does not change the number of feature map channels. They are only used in the backbone of the network to extract features.

To further reduce the computation of the network, we also design a new residual module to replace the two CBL modules in CSP2_X. We name the proposed residual unit and improved CSP2_X as ResNet2 unit and ICSP2_X, respectively. The ResNet2 unit and ICSP2_X are shown in Figure 1. The proposed ResNet2 unit also contains two branches. The upper branch includes a CBL module, a DWCBL module, and a 1 × 1 convolution. The CBL module consists of a 1 × 1 standard convolution, a BN module, and a LeakyReLU activating function. The CBL module is used to increase the depth of the network, which is useful for extracting more detailed information about the defective insulator. The number of output channels for the CBL module is half the number of input channels in the ResNet2 unit. The DWCBL module consists of 3 × 3 depthwise separable convolutions, a BN module and a LeakyReLU activating function. The input feature map of DWCBL module has the same number of channels as the output feature map of DWCBL module. The last 1 × 1 standard convolution in ResNet2 unit is also used to increase the network depth. The lower branch only consists of a 1 × 1 standard convolution. The number of output channels for the 1 × 1 standard convolution is half the number of input channels. In the end, the upper and lower branches are connected by concatenating operation to construct the complete ResNet2 unit. The input feature map of the proposed ResNet2 unit module has the same number of channels as the output feature map of the ResNet2 unit. We use the proposed ResNet2 unit to replace two CBL modules in the original CSP2_X to obtain the ICSP2_X module. TheICSP2_X also consists two branches that are named upper branch and lower branch, respectively. The upper branch consists of a CBL module, *X* ResNet2 unit and a 1 × 1 standard convolution. The number of output channels for the upper branch is also the is half the number of input channels. The lower branch of ICSP2_X is only a 1 × 1 standard convolution to reduce the number of input channels. The concatenating operation is also used to connect upper and lower branches. The same BN module, LeakyReLU activating function, and CBL module are also used to extract features from the concatenated feature map in the ICSP2_X module. Besides, we also introduce the channel shuffle into ICSP2_X to facilitate the flow of feature information from different channels.

In the ICSP1_X and ICSP2_X, we use depthwise separable convolution to replace the standard 3 × 3 convolution. The FLOPs of standard convolution can be expressed as:

(1) $$\begin{array}{c} N_{c}=2\times K^{2}\times C_{in}\times C_{out}\times H\times W, \end{array}$$

where *K* is the kernel size of standard convolution, *C*_*in*_ and *C*_*out*_ are the number of channels for input and output, respectively, and *H*and *W* are the width and height of the feature map. The FLOPs of depthwise separable convolution can be expressed as:

(2) $$\begin{array}{c} D_{c}=2\times \left(K^{2}\times C_{in}+C_{out}\times C_{in}\right)\times H\times W. \end{array}$$

The FLOPs ratio of standard convolution and depthwise separable convolution is:

(3) $$\begin{array}{c} \frac{D_{c}}{N_{c}}=\frac{2\times \left(K^{2}*C_{in}+1^{2}*C_{in}*C_{out}\right)*H*W}{2\times K^{2}*C_{in}*C_{out}*H*W}\\ =\frac{K^{2}+C_{out}}{K^{2}*C_{out}}. \end{array}$$

In YOLO5s, the numbers of output channels are 128, 256, and 512 for different modules. The kernel sizes are 1 or 3 for other convolutions. The number of output channels is much larger than kernel size, so the (3) can be simplified as:

(4) $$\begin{array}{c} \frac{D_{c}}{N_{c}}=\frac{K^{2}+C_{out}}{K^{2}*C_{out}}\approx \frac{C_{out}}{K^{2}*C_{out}}=\frac{1}{K^{2}}. \end{array}$$

The original ResNet unit of CSP1_X contains a 1 × 1 convolution and a 3 × 3 convolution. The proposed ResNet unit of ICSP1_X contains two 1 × 1 convolutions and two 3 × 3 depthwise separable convolutions. For the CSP1_X, the computation of the proposedResNet1 unit and original ResNet unit is about 2/9. For the CSP2_X, the computation of the proposed ResNet2 unit and CBL model is about 1/9. Our proposed two networks that are the ResNet1 unit and RestNet2 unit reduce the computation of CSP1_X and CSP2_X. The proposed ICSP1_X and ICSP2_X have smaller computations than the original CSP1_X and CSP2_X.

### 3.2. Separate Embedded Coordinated Attention Module

**Figure 2:**
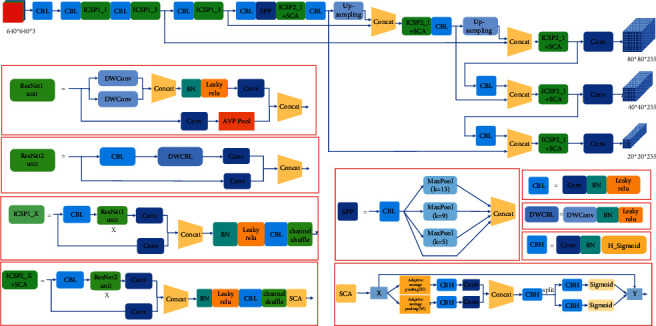
Improved YOLOv5s.

The backbone of our improved YOLOv5s based on our proposed ICSP1_X and ISCP2_X has few parameters and computation but also reduces feature extraction ability. Inspired by [37], to balance the detection speed and precision, we designed a separated embedded coordinated attention module named SCA module and introduce it into ICSP1_X module and ICSP2_X module to extract more useful defective insulator features. The proposed SCA module is shown in Figure 2. It consists of two branches: the upper branch consists of short cut to reserve input feature map. The lower branch is used to compute the weight of useful features; the lower branch has two average pooling operations: adaptive average pooling (H) and adaptive average pooling (*W*). The kernel sizes of adaptive average pooling (H) and adaptive average pooling (*W*) are *H* × 1 and 1×*W*, respectively. They encode each channel along the horizontal and vertical coordinates, respectively. We can obtain two separate position-aware feature maps from the two adaptive average pooling operations. Therefore, we can capture long-range dependencies of feature information along one spatial direction while retaining precise location information in the other spatial direction. To make the network have better expression ability. We use CBH modules consisting of a convolution layer, a BN layer and a H_sigmoid activate function. Without changing the size of the direction-aware feature map, we use the 1 × 1 convolution layer to increase the depth of the network, use BN layer to improve the generalization ability of the network and use the H_sigmoid active function to increase the nonlinear representation ability of the network. Then two 1 × 1 convolutions are used to reduce the number of channels to half the original number. The output feature of two branches that contain two adaptive average pooling are connected by concatenate operation. The CBH module is also used to fuse the feature obtained by concatenate operation. The split method is used to separate the feature map from the horizontal coordinate and vertical coordinate. In the end, we can obtain the weight of the horizontal coordinate, and the weight of the vertical coordinate of the feature map by the CBH module and sigmoid activate function. We multiply the input feature map by two weights to get the output feature map.

**Figure 3:**
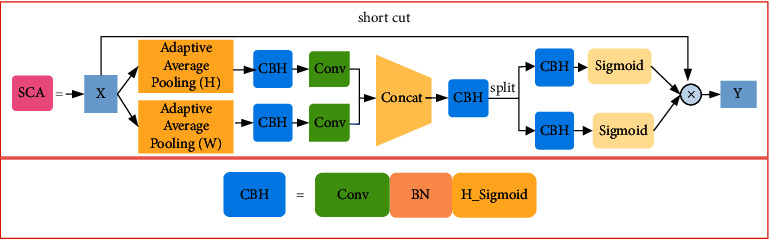
Proposed separate embedded coordinated attention module.

To better explain the model, we suppose that the input feature map *X* ∈ *R*^*C*×*H*×*W*^. The number of channels is *C* , and the size of the feature map is *H* × *W*. Output feature map of adaptive average pooling (H) and adaptive average pooling (*W*) in the *cth* channel can be expressed as *Z*_*c*_(*H*) and *Z*_*c*_(*W*). They can be expressed as followings:

(5) $$\begin{array}{c} Z_{c}\left(H\right)=\frac{1}{W}\underset{0\leq i<W}{\sum }X_{c}\left(H,i\right)\\ Z_{c}\left(W\right)=\frac{1}{H}\underset{0\leq i<H}{\sum }X_{c}\left(i,W\right)\text{,} \end{array}$$

where *X*_*c*_ is the input feature map in the *cth* channel. The sizes of *Z*_*c*_(*H*) and *Z*_*c*_(*W*) are *H* × 1 and 1 × *W*, respectively. The 1 × 1 convolution is used to halve the number of output channels of CBH in Figure 3. The concat operation connects the two output feature maps obtained from CBH and a 1 × 1 convolution. It can be expressed as follows.

(6) $$\begin{array}{c} X_{1}=\left[f^{1\times 1}\left(CBH\left(Z\left(H\right)\right)\right),f^{1\times 1}\left(CBH\left(Z\left(W\right)\right)\right)\right], \end{array}$$

where *f*^1×1^ is the 1 × 1 convolution, [, ] is the concatenating operation. *X*_1_ is the output feature map of concat operation in Figure 2. Next, we use the split function to separate the output feature of the CBH module that is connected with concat operation module into two parts. In the end, we obtain the weights of two parts. The sizes of weight are *H* × 1 and 1 × *W*, respectively. The size of the final weight obtained by multiplying two weight vectors is *H* × *W*. Finally, we multiply the input feature map *X* with the final weight to get the output feature map.

### 3.3. Improved Focus Module

In YOLOv5s, it uses focus to realize down-sampling without losing information. In focus, it firstly uses a slicing operation to expand the input channel four times. Second, it uses a 3 × 3 convolution to obtain a down-sampling feature map. There is a large computation for the slicing operation. To reduce computation, we use a 3 × 3 convolution with a down-sampling function to replace the focus module. We suppose the input image size is 640 × 640 × 3 and the size of the output feature map size is 320 × 320 × 32. The FLOPs of focus and convolution without considering the BN and activate function are shown in (7) and (8), respectively.

(7) $$\begin{array}{c} Focus=3\times 3\times 12\times 32\times 320\times 320=353894400. \end{array}$$

(8) $$\begin{array}{c} Cov=3\times 3\times 3\times 32\times 320\times 320=88473600. \end{array}$$

Based on (7) and (8), the computation of the focus model is about four times larger than convolution. Therefore, using convolution to replace focus can reduce computation.

Based on our proposed ICSP1_X, ICSP2_X, SCA, and improved focus modules, the improved YOLOv5s are shown in Figure 2. The pseudo-code of our proposed method is shown in Table 1. The Adam optimizer method is used to optimize the parameters of the network.

## 4. Simulation and Discussion

The experimental environment is configured as follows: the operating system is Ubuntu 18.04. The deep learning framework is Pytorch. Six detection methods that our proposed method, YOLOv3 [10], YOLOv4 [11], YOLOv5s [9], Fast R-Transformer [38], and Mina-Net [31] method are set with the same parameters and pre-training weights are used. The initial learning rate was set to 0.001, and the learning rate was dynamically adjusted using the cosine annealing learning rate. To prevent overfitting, the Adam optimizer is used to adjust the parameters. Pre-training is used in the first five epochs to accelerate the convergence of the model. Pre-training weights are used for all the above methods.

**Figure 4:**
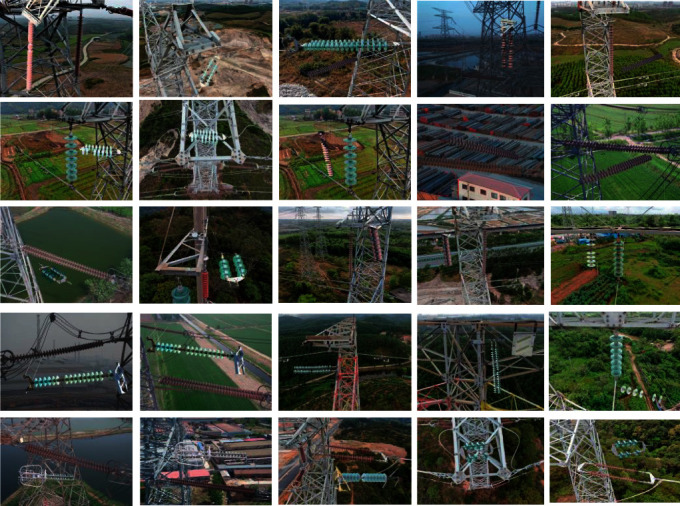
Parts of generated defective insulator images.

We sourced 195 insulator images from the Power supply and the Internet. The number of defective insulator images is too small to train the network. Therefore, we use the same method used in the CPLID dataset [39] to expand the data set. We extract defective insulators and fuse them with different backgrounds. The parts of generated defective insulator images are shown in Figure 4. Our proposed defective dataset contains 2300 images. We use 1800 images as training images and 500 images as test images.

### 4.1. Ablation Study

We design a new ResNet1 unit and ResNet2 unit and introduce them into CSP1_X and CSP2_X modules to reduce the computation. Besides, we also introduce the channel shuffle operation into improved CSP1_X and CSP2_X modules. To simplify, we name the YOLOv5s based on improved CSP1_X and CSP2_X without channel shuffle operation as YOLOv5s + Res, YOLOv5s based on improved CSP1_X and CSP2_X with channel shuffle operation as YOLOv5s + Res + Cs. Besides, we also design a separate embedded coordinate attention module and introduce it into the improved and CSP2_X modules. We name the YOLOv5s + Res + Cs based on a separate embedded coordinate attention module as YOLOv5s + Res + Cs + SA. We also name the YOLOv5s + Res + Cs based on the convolutional block attention module [40] as YOLOv5s + Res + Cs + CBAM, and based on Squeeze-and-Excitation [41] as YOLOv5s + Res + Cs + SE. In addition, we also use convolution to replace focus in YOLOv5s. The YOLOv5s + Res + Cs + SA based on a convolution without using focus is named YOLOv5s + Res + Cs + SA-Foc, which is also our complete improved YOLOv5s. The results are shown in Table 2.

The YOLOv5s have the most parameters, FLOPs, and the highest mean average precision (mAP). The YOLOv5s + Res that YOLOv5s based on improved CSP CSP1_X and CSP2_X have the smallest parameters, FLOPs, and mAP. As the number of parameters decreases, the mAP also decreases. Although the YOLOv5s + Res + cs that YOLOv5s based on improved CSP CSP1_X and CSP2_X with channel shuffle operation have the same number of parameters and FLOPs, but it has larger mAP than YOLOv5s + Res. The channel shuffle operation improves the precision without increasing parameters and FLOPs. Compared with YOLOv5s + Res + Cs, the YOLOv5s + Res + Cs + SE, YOLOv5s + Res + Cs + CBAM and YOLOv5s + Res + Cs + SA that are introduced different attention modules into YOLOv5s + Res + Cs have larger parameters, FLOPs, and mAP. The attention mechanism improves detection precision while increasing parameters and FLOPs. Compared with YOLOv5s + Res + Cs + SE and YOLOv5s + Res + Cs + CBAM, YOLOv5s + Res + Cs + SA has the larger mAP. The SA module is our proposed attention module. This shows that our proposed attention module performs better in precision than SE and CBAM attention modules. Compared with YOLOv5s + Res + Cs + SA, the YOLOv5s + Res + Cs + SA-Foc that uses convolution to replace the focus module to reduce computation has smaller parameters, FLOPs, and mAP.

We propose the improved CSP1_X module and CSP2_X module and modify the focus module to reduce computation. We propose an attention module and introduce it into the network to improve precision. Based on the analysis of Table 2, the improved CSP1_X module, CSP2_X module, and modified focus reduce the parameters and FLOPs, and the proposed attention module increases precision.

### 4.2. Comparisons for Different Methods

**Figure 5:**
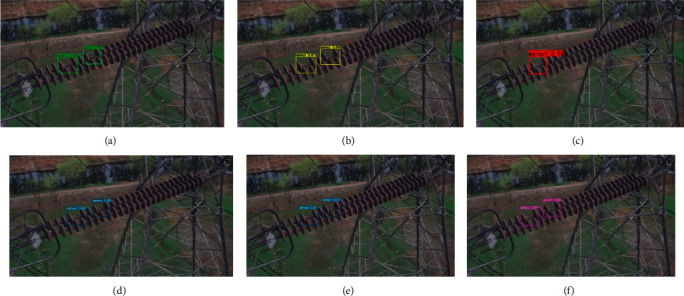
Defective porcelain insulator detection.

**Figure 6:**
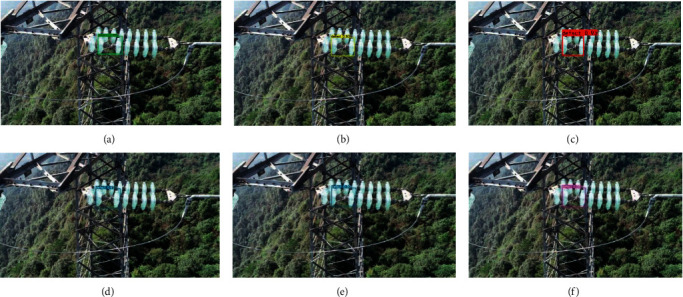
Defective toughened glass insulator detection.

**Figure 7:**
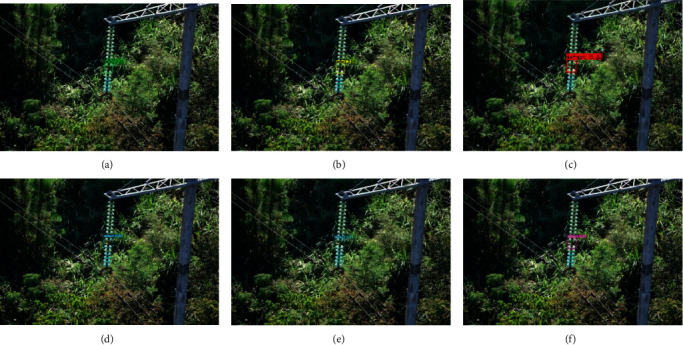
Defective suspended glass insulator detection.

We compare our complete proposed method with YOLOv3, YOLOv4, and YOLOv5s, Fast R-Transformer, and [38] Mina-Net [31]. We randomly select three different insulators that contain defective insulators. The detection results are shown in Figures 5–7. In each figure, figure (a), figure (b), figure (c), figure (d), figure (e), and figure (f) are obtained by Fast R-Transformer, Mina-Net YOLOv3, YOLOv4, YOLOv5s, and our proposed method, respectively. In Figure 5, there are two defective insulators, and the YOLOv3 only detects one defective insulator. The YOLOv4, YOLOv5s, Fast R-Transformer, Mina-Net, and our proposed method successfully detect all defective insulators. In Figures 6 and 7, all methods successfully detect all defective insulators. This shows that the proposed method is valid for defective insulator detection.

To verify the performance of the proposed method from a statistical point of view, we use YOLOv3, YOLOv4, YOLOv5s, Fast R-Transformer, Mina-Net, and our proposed to detect all images in the test dataset. The detection results are shown in Table 3. The parameters of Fast R-Transformer, Mina-Net, YOLOv3, YOLOv4, YOLOv5s, and our proposed method are 116.10 M, 67.94 M, 61.90 M, 63.94 M, 7.06 M, and 5.45 M, respectively. Our proposed method has the smallest parameters, followed by YOLOv5s. The FLOPs of Fast R-Transformer, Mina-Net YOLOv3, YOLOv4, YOLOv5s, and our proposed method are 257.5 G, 168.2 G, 155.1 G, 157.1 G, 16.3 G, and 12.5 G, respectively. Our proposed method still has the smallest parameters, followed by YOLOv5s. The mAPs of Fast R-Transformer, Mina-Net, YOLOv3, YOLOv4, YOLOv5s, and our proposed method are 99.7%, 99.2%, 91.5%, 98.7%, 94.5%, and 93.6%, respectively. The Fast R-Transformer has the largest mAP, followed by Mina-Net and YOLOv4. The FPS of Fast R-Transformer, Mina-Net, YOLOv3, YOLOv4, YOLOv5s, and our proposed method are 38.6, 47.5, 54.6, 52.7, 157.4, and 197.8, respectively. Our proposed method has the largest FPS, followed by YOLOv5s.

Based on the analysis of Table 3, our proposed method has the smallest parameters, FLOPs, and the largest FPS. The FLOPs of different models are computed by model_into() function in touch_utils.py. Compared with YOLOv5s, although the mAP of our proposed method is reduced by 0.9%, FPS of our proposed method is increased by about 20%. Although our proposed method has a smaller mAP than other methods, our proposed method has a faster detection speed. Besides, the increase in detection speed is greater than the decrease in precision.

## 5. Conclusions

This study proposed a faster defective insulator detection method based on YOLOv5s. It designs two different ResNet units for CSP1_X module and CSP2_X module in YOLOv5s to reduce computation, respectively. It also introduced channel shuffle into CSP1_X module and CSP2_X module to extract more effective features from different channels without increasing computation. Besides, it designed a separate embedded coordinated attention module and introduced it into the CSP2_X module to make the network pay more attention to useful information. To reduce the computation, it replaced the focus module using a convolution with stride 2. Compared with defective insulator detection methods based on Fast R-Transformer, Mina-Net, YOLOv3, YOLOv4, and YOLOv5s, our proposed method has the smallest parameters and FLOPs and the largest FPS. This shows that the proposed method has the fastest defective insulator detection speed. Although the other methods except YOLOv3 have larger mAP than our method, the difference in mAP is not large. The mAP of Fast R-Transformer is the largest. Although the mAP of Fast R-Transformer is 6.1% higher than that of our method, the detection speed of Fast R-Transformer is only 1/5 of our method detection speed. Our method and YOLOv5s have a faster detection speed than others. Compared with YOLOv5s, although the mAP of our proposed method is reduced by 0.9%, the detection speed of our proposed method is increased by about 20%. The increase in detection speed is greater than the decrease in precision. The proposed method has a faster detection speed and an acceptable detection precision.

## Figures and Tables

**Table 1:** Pseudo code of our proposed method.

$\overset{⌢}{\theta }\leftarrow Improved\;YOLOv5sTraning\left(z_{1:N_{data}},x_{1:N_{data}},\theta \right)$
/^∗^Training network model
Input: {(*z*_*n*_, *x*_*n*_)}_*n*=1_^*N*_*da* *ta*_^, a dataset of sequence pairs
Input:*θ*, initial model parameters
Output: $\overset{⌢}{\theta }$, the trained parameters
Hyperparameters: *N*_*epochs*_ ∈ N
For *i*=1,2, ⋯, *N*_*epochs*_ *do*
For *n*=1,2, ⋯, *N*_*da* *ta*_ *do*
*θ* ← Improved YOLOv5s((*z*_*n*_, *x*_*n*_, *θ*))
$\begin{array}{c} loss\left(\theta \right)\leftarrow l_{con}\left(\theta \right)+0.5\times l_{cls}\left(\theta \right)+\\ \;0.05\times l_{box}\left(\theta \right) \end{array}$
$\begin{array}{c} m_{n}\leftarrow \beta_{1}m_{n-1}+\left(1-\beta_{1}\right)\nabla loss\left(\theta \right)\\ v_{n}\leftarrow \beta_{2}v_{n-1}+\left(1-\beta_{2}\right)\nabla loss{\left(\theta \right)}^{2}\\ {\overset{⌢}{m}}_{n}\leftarrow \frac{m_{n}}{1-\beta_{1}},{\overset{⌢}{v}}_{n}\leftarrow \frac{v_{n}}{1-\beta_{2}}\\ \theta \leftarrow \theta -\frac{\mu }{\sqrt{{\overset{⌢}{v}}_{n}}+\epsilon }\times {\overset{⌢}{m}}_{n} \end{array}$
End
End
Return $\overset{⌢}{\theta }=\theta$

**Table 2:** YOLOv5s with different modules.

Methods	Params (M)	FLOPs (G)	mAP (%)
YOLOv5s	7.06	16.3	94.5
YOLOv5s + Res	5.19	12.2	91.7
YOLOv5s + Res + Cs	5.19	12.2	93.2
YOLOv5s + Res + Cs + SE	5.38	12.3	93.5
YOLOv5s + Res + Cs + CBAM	5.40	12.4	93.7
YOLOv5s + Res + Cs + SA	5.47	12.8	94.1
YOLOv5s + Res + Cs + SA-foc(ours)	5.45	12.5	93.6

**Table 3:** Comparisons for different methods.

Methods	Params (M)	FLOPs (G)	mAP (%)	FPS
Fast R-Transformer	116.1	257.5	99.7	38.6
Mina-net	67.94	168.2	99.2	47.5
YOLOv3	61.90	155.1	91.5	54.6
YOLOv4	63.94	157.1	98.7	52.7
YOLOv5s	7.06	16.3	94.5	157.4
Ours	5.45	12.5	93.6	197.8

## Data Availability

The labeled dataset used to support the findings of this study is available from the corresponding author upon request.
